# Formation and Characterization of Hole Nanopattern on Photoresist Layer by Scanning Near-Field Optical Microscope

**DOI:** 10.3390/nano9101452

**Published:** 2019-10-12

**Authors:** Agata Roszkiewicz, Amrita Jain, Marian Teodorczyk, Wojciech Nasalski

**Affiliations:** 1 Institute of Fundamental Technological Research, Polish Academy of Sciences (IPPT PAN), Adolfa Pawińskiego 5b, 02-106 Warsaw, Poland; arosz@ippt.pan.pl (A.R.); ajain@ippt.pan.pl (A.J.); wnasal@ippt.pan.pl (W.N.); 2 Institute of Electronic Materials Technology (ITME), Wólczyńska 133, 01-919 Warsaw, Poland; marian.teodorczyk@itme.edu.pl

**Keywords:** optical lithography, photoresist, quartz, hole nanopatterning

## Abstract

Patterning of lines of holes on a layer of positive photoresist SX AR-P 3500/6 (Allresist GmbH, Strausberg, Germany) spin-coated on a quartz substrate is carried out by using scanning near-field optical lithography. A green 532 nm-wavelength laser, focused on a backside of a nanoprobe of 90 nm diameter, is used as a light source. As a result, after optimization of parameters like laser power, exposure time, or sleep time, it is confirmed that it is possible to obtain a uniform nanopattern structure in the photoresist layer. In addition, the lines of holes are characterized by a uniform depth (71–87 nm) and relatively high aspect ratio ranging from 0.22 to 0.26. Numerical modelling performed with a rigorous method shows that such a structure can be potentially used as a phase zone plate.

## 1. Introduction

Photolithography is considered a dominant method for patterning nanoscale features in the microelectronics industries. In the typical process of photolithography, a mask is necessary because it carries the information of the desired pattern, which is transferred by using a suitable optical technique. Nowadays, advanced nanolithography techniques are attracting researchers as well as industries because of their capability of providing shrinking feature sizes as well as increased integration density [1,2,3,4]. Since optical diffraction imposes an ultimate limit on the minimum feature size that can be fabricated by using optical lithography, alternative approaches, such as wet lithography; unconventional lithography [5,6]; or scanning probe microscope (SPM)-based lithography, also known as scanning probe lithography (SPL), have received much attention in nanopatterning [1,7,8]. The attractiveness of the SPL approach is caused by the fact that these methods can successfully overcome the Rayleigh limit, as they operate in the near-field range. Different types of SPL techniques, such as atomic force microscope (AFM) [9,10,11,12,13], scanning tunneling microscope (STM) [14], and scanning near-field optical microscope (SNOM) [15,16,17,18] have been studied and modified, and their applicability for nanofabrication has been reported. The prime advantage of using the SPL technique is its ambient working conditions, as all these methods can be exercised in air medium and at room temperature, whereas techniques like electron beam lithography (EBL) should be performed in vacuum. Moreover, the resolution of SPL has also been significantly improved, and it is almost close to that obtained by EBL [12,19,20]. SPL methods are also preferred, as they facilitate the direct-writing method and do not need a separate mask for nanopatterning of samples. Mask fabrication, especially in small sizes, is a costly and time-consuming process, hence SPL appears overall to be a cost-effective and time-saving procedure. However, the inherent disadvantage of using any kind of SPL technique is its slow scanning speed, which results in the slow process of structuring, which makes this method unsuitable for mass production. However, this approach is certainly profitable for the fabrication of samples in the research scale [21,22]. In order to overcome this constraint, parallel nanoprobes can be used to increase productivity [23,24].

Among all the SPM techniques used for lithography, the most popular technique is SNOM, which was first introduced by Synge [25]. It is preferred mostly because it can be applied to existing resist techniques, and it also withstands the requirements of recently introduced novel materials [17,26]. Moreover, scanning near-field optical lithography (SNOL) also satisfies the increasing demand of inexpensive lab tools to prepare and manipulate nanostructures of dimensions smaller than 100 nm. Near-field light, which is generated at the tip of the probe, is used for the exposure of photoresist during scanning. SNOL has been successfully used for the patterning of different organic materials like positive photoresist [27,28]; negative photoresist [29]; polymethylmethacrylate (PMMA) resist [17,26]; conjugated polymers [16,30] and azopolymer films [31,32]; and inorganic materials like metals [33,34], H-passivated Si [35], etc.

In this article, we report on the patterning of lines of holes carried out on positive photoresist by using SNOM. Photoresist was deposited on quartz substrate by using spin coating method. Nd:YAG laser with wavelength 532 nm was used as a power source. The present work mainly focuses on using the non-standard configuration of laser, photoresist, and structure, but is still suitable for photonic applications. This allows one to go beyond the high absorption part of the photoresist spectrum, as the maximum absorption and short exposure times should not always be the main focus of experiments of this type.

In [18,36,37], the authors used lasers of wavelengths in the high absorption region of the photoresist spectrum (450 nm, 442 nm, and 400 nm, respectively). They obtained SNOL patterns in photoresist that were characterized by rather small depths and moderate aspect ratios (the ratio of depth and Full Width of Half Maximum (FWHM) of the groove). Our paper is the continuation of their work. We intend to solve the same problem with a slightly different method. First, we use the longer-wavelength laser (532 nm); second, we focus on holes instead of grooves. We have choose a laser wavelength that lies in the less sensitive part of the spectrum, because we expect that some parameters of the structure obtained at this wavelength will be better than those at the maximum absorption wavelength. In addition, the use of holes instead of grooves increases the number of parameters that can be optimized and thus may lead to flexibility in the interaction of design and enriched light-matter, which is of importance in nanophotonic applications. As will be presented in Section 3.3, a focusing structure made of holes is characterized by a significantly shorter focus than this in the case of grooves. While performing the experiments, parameters like laser power, exposure time, and sleep time are optimized for the present configuration (photoresist|substrate|laser), and changes in width and depth with respect to the exposure time are also examined.

The main goal of this work is using a laser of wavelength that lies beyond the high absorption part of the photoresist spectrum in order to search for other experimental conditions suitable in forming various photonic structures. The article is divided into four sections: materials and methods, used to prepare the sample, followed by photolithographic experiment description, results and discussions, and final conclusions.

## 2. Materials and Methods

### 2.1. Preparation of Substrates

In this study, ST-cut monocrystalline doubly-polished, 500 µm-thick quartz wafer was used. Square substrates of 15 × 15 mm were cut from 4′′ wafer with a diamond blade installed in a DISCO DAD 2H 6T saw (DISCO, Tokyo, Japan). A positive photoresist SX AR-P 3500/6 (Allresist GmbH, Strausberg, Germany) was used. Since it contains novolac resins combined with a special light sensitive component, it is more than usually sensitive in the long wavelength visible range. The absorption spectra of the photoresist is discussed in detail in the third section. After patterning of the substrate, AR 300-26 (1:2 diluted with deionized water (DI)) developer (Allresist GmbH, Strausberg, Germany) was used to develop the samples for 60 s followed by washing with DI and drying with pure nitrogen gas. Finally, the samples were hard baked at 100 °C for 2 min.

For the sample preparation, standard RCA cleaning procedure was performed. In the beginning, samples were cleaned in ultrasonic bath for the removal of particles that could be removed in standard cleaning process. Next, the substrate was placed into a hot trichloroethylene (TCE) solution (to eliminate organic residues), acetone (to remove TCE residues), isopropyl alcohol (IPA) (to remove acetone residues), and DI (to remove IPA residues). In the next step, samples were put into concentrated sulfuric acid (H_2_SO_4_) at 70 °C followed by washing in flowing DI water. After that, the samples were soaked in SC1 solution (NH_4_OH:H_2_O_2_) and rinsed with DI water. In the next step, the samples were put into SC2 (HF:H_2_O) solution and finally washed with DI water, blow dried with high-purity nitrogen gas, and dried for several hours in vacuum chamber at 200 °C. After that, the surface was activated with plasma etching using argon plasma. After above steps, a thin layer of diluted (diluted by using EC-SOLVENT, Microposit, Micro resist technology GmbH, Berlin, Germany) photoresist SX AR-P 3500/6 was deposited by using LSI 445/60 centrifuge (EATON, Tinton Falls, NJ, USA) in semi-automatic processes. The samples were spin coated for 30 s. After spin coating, the samples were dried in the oven at 90 °C for 20 min. Dilution for this photoresist is required, as the reflected light from the substrate affects the desired pattern, which limits the thickness of the photoresist [38].

To obtain the calculated thickness of the photoresist, it was necessary to experimentally select the dilution and the speed of the rotation during the coating. The final thickness of the photoresist was 700 nm, and it was measured by using profilometer VEECO DEKTAK 150 (Aschheim/Dornach Munich, Germany).

### 2.2. Scanning Near-Field Optical Lithography System

**Figure 1:**
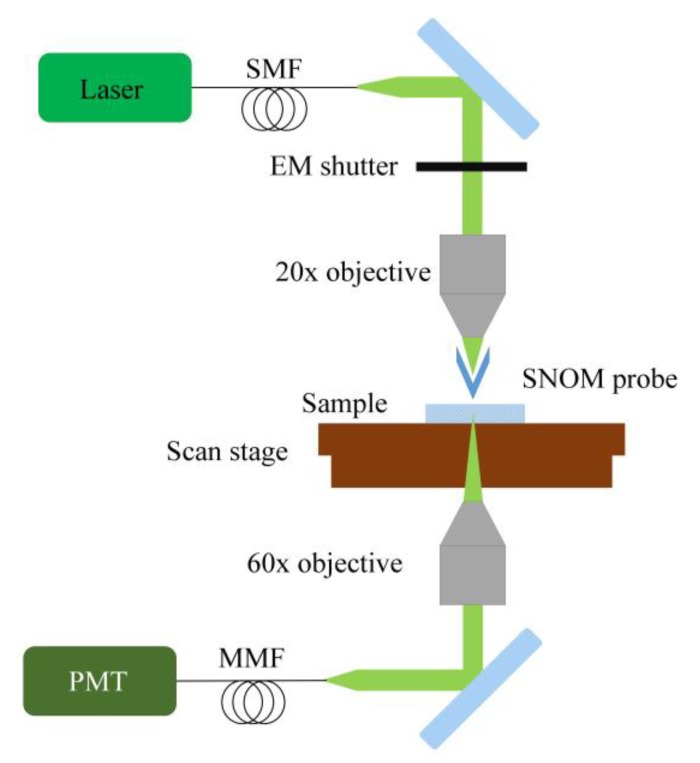
A schematic representation of the SNOM experimental setup: single mode fiber (SMT), multimode fiber (MMF), and photomultiplier tube (PMT).

A schematic representation of the experimental setup is shown in Figure 1. The alpha300 S SNOM (WITec GmbH, Ulm, Germany) was used to perform the experiment. The SNOM was operated in contact mode during patterning. A frequency doubled continuous wave Nd:YAG laser (wavelength 532 nm, maximum output power 70 µW, WITec GmbH, Ulm, Germany) was used as the light source. The laser beam was coupled with a single mode optical fiber and focused by using 20× objective lens on the back side of the probe. The high-quality, micro-fabricated SNOM sensor had a shape of a hollow aluminum pyramid and was mounted on a silicon cantilever. The diameter of the aperture of the tip was 90 nm, with a height of 15 µm and a base of 20 × 20 µm^2^, which gave approximately 65° of a cone angle. Since the skin depth of the aluminum was very small at this wavelength (few nm), the light transmission through the coating layer of the probe could be neglected. The position of the probe with respect to the sample surface was controlled by an optical feedback system, in which the infrared laser beam reflected from the cantilever was monitored with a photodiode. For automated control of patterning, an electromagnetic shutter obtained from SHB05, ThorLabs, Newton, NJ, USA was used.

The photochemical reactions occurring in the photoresist under the exposure light depended on the total amount of absorbed energy. During the SNOL action, the sample was exposed to an evanescent light coming from a subwavelength aperture at the tip of the probe. Due to the wide opening angle and the specific structure, the transmission coefficient of the tip used (defined as the ratio of the transmitted and incident power) was much higher than this in case of fiber probes of the same diameter [39]; however, it was still around 10^−3^ [40].

## 3. Results and Discussions

The following section is divided into three parts, in which absorption characteristics of photoresist, pattern characterization, and numerical modeling of optical field focusing of the obtained structure are discussed.

### 3.1. Absorption Spectra of Photoresist

**Figure 2:**
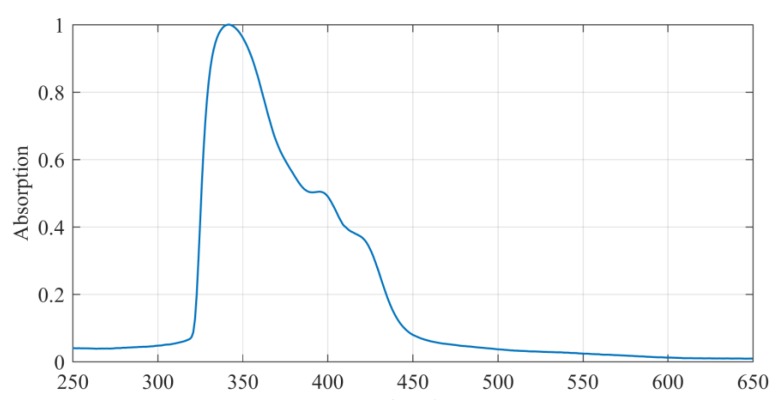
Absorption spectrum of SX AR-P 3500/6 photoresist. Spectra of the standard positive photoresists are available on Allresist’s page [41].

Spectral absorbance of SX AR-P 3500/6 photoresist is shown in Figure 2. The spectrum was measured by using FS5 Spectrophotometer, Edinburgh Instruments, on unexposed photoresist. As can be seen from the figure, the absorption of this photoresist, although custom-made, is most effective in ultraviolet light and it is not very sensitive at 532 nm. As a result, laser power during photolithography is increased to achieve the required energy dose. However, when the probe is subjected to high laser power for a long time, it heats up and generates heating effects on the photoresist below. In extreme cases, the metal coating of the probe can be also partially melted. After optimization process, the laser power (measured at the exit of the laser chamber) was set to 2 µW. The distance between the tip and the sample is one of the major factors that influences the dimensions of the final structure. Since the field is evanescent, it can affect the photoresist only if the distance is below the wavelength distance (near-field). The varied distance between the tip and sample was used before to control the dimensions of the peak evanescent energy spot by use of feedback loop [18]. In the present studies, the cantilever was slightly retracted from the surface before patterning to avoid the local heating effects. In addition, sleeping time was also introduced after each hole, during which the system automatically closed the laser shutter and allowed the probe to cool down.

### 3.2. Characterization of the Obtained Pattern

**Figure 3:**
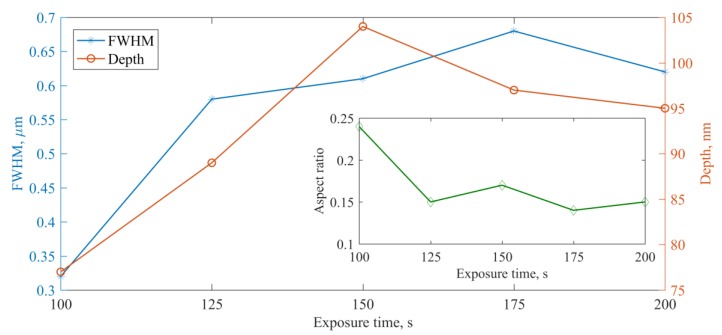
Variation of Full Width of Half Maxium (FWHM) and depth of the holes with respect to exposure time at constant laser beam power. Inset shows the variation of aspect ratio with respect to exposure time.

Figure 3 represents the dependence of parameters like width and depth of holes on exposure time. This experiment was carried out mainly to determine the maximum width and depth of holes, when exposure time increased. As can be seen from the figure, width and depth increase with increasing exposure time. The depth increase for longer exposure times is not significant; rather, it is saturated. However, width increase results in decreasing the visual resolution of the structure. Inset of Figure 3 shows the variation of aspect ratio with exposure time, and it can be clearly seen that for the structure having exposure time of 100 s the aspect ratio is noticeably higher compared with other cases. Hence, for detailed examination of the structure 100 s exposure time was chosen as it shows better resolution with higher aspect ratios at less exposure time.

**Figure 4:**
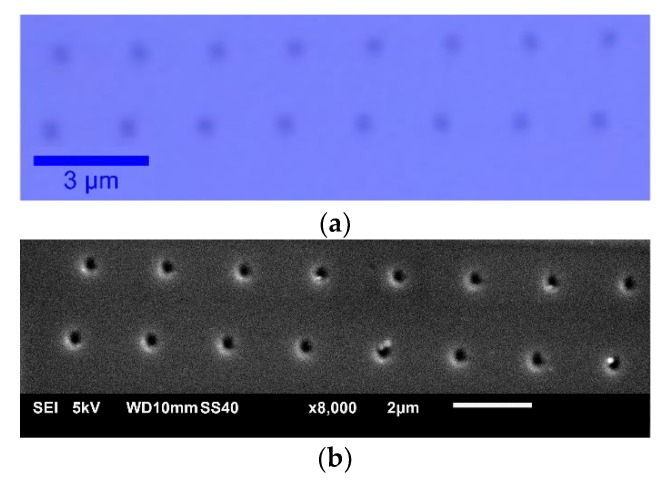
Pattern visualization with (**a**) confocal 100x and (**b**) scanning electron microscopy.

The preliminary visualization of the structure was performed by using our microscope in a confocal mode. Figure 4a represents the typical test structure in confocal view with 100× magnification. The holes are regular and seem to have uniform depth. However, the contrast and resolution of the confocal microscope mode is limited, so to assess the quality of the pattern, scanning electron microscope (SEM) was used. The scanning was performed with JEOL JSM-6010Plus/InTouchScope (JEOL, Tokyo, Japan). Figure 4b shows the SEM image of the final pattern on the photoresist after its development. Approximately 2–5 nm thick gold layer was deposited on the sample prior to the experiment. It can be clearly seen from the figure that lines of the holes resemble each other in shape and local orientation and size of the holes are very uniform. This is also confirmed from AFM analysis, which is discussed further in this section.

**Figure 5:**
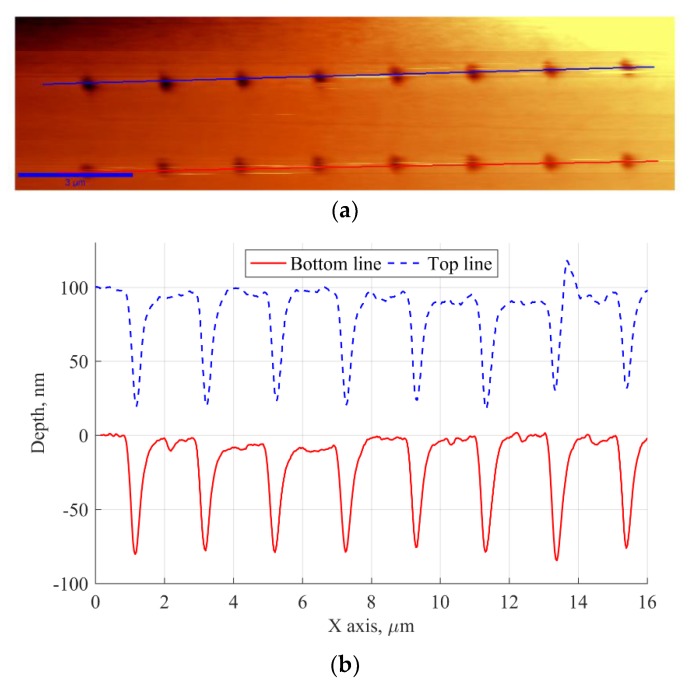
(**a**) AFM scan topography of the optimized structure and (**b**) cross sectional profile of the holes along the marked lines of the optimized pattern at constant laser beam power.

Figure 5a,b shows an AFM topography scan of the structure and the linear cross sections of the holes in both lines. The contact mode AFM scanning was used and the studies was performed by using alpha SNOM 300 S with use of aluminum-coated AFM cantilever with force constant equal to 0.2 N/m procured from WITec GmbH.

The repeatability and uniformity of the pattern in the photoresist depend on many factors. It is difficult to reproduce the same pattern with different probes or even with the same probe but with new alignment [36]. The magnetic connections between the cantilever and an arm and between the arm and the motorized stage with the objective might cause slight displacements while the tip is approaching the sample. Even a small change of precise microalignment may result in significantly changed optical throughput of the probe. Besides, a new sample, ambient conditions, slightly modified laser power, etc., are factors that might decrease the reproducibility [36]. However, the presented hole array structure was made in constant conditions and during precise alignment of the probe. In effect, the shape and depth of the holes in the whole pattern shown are quite regular.

It is worth stressing that the depths of the holes, evaluated with AFM, range from 71 to 87 nm. The dominant value of FWHM of the hole cross-section is 320 nm, with minimum and maximum values of 300 nm and 340 nm, respectively. Hence, despite the small retraction of the tip, hole array pattern with FWHM about 0.56–0.64 wavelength was obtained. Since low laser power requires longer exposure times to achieve the same amount of exposure dose in the photoresist, the exposure time of each hole was set to 100 s. After the exposure of each hole, the shutter was closed; the probe was then moved half the way to the next hole at fixed vertical distance from the sample, cooled down for 100 s, and moved again half way to expose the next spot. It is noteworthy that there was no trace of exposure between the holes, which confirms that the pattern was made with an evanescent light and no heat came from the probe.

According to the Bethe–Bouwkamp model [42,43], the evanescent field emerging from the tip has a Gaussian distribution. At short subwavelength distances, the evanescent field dominates and allows one to obtain small-size patterns, unlimited by far field diffraction. Aghaei et al. [18] shows that low scanning speeds (and in extreme cases, constant exposure in one spot) may lead to a very low resolution due to a Gaussian profile of the evanescent field (for example, with scanning speed of 1 µm/s they obtained line width of 511.7 nm, while with 50 µm/s speed resulted caused the line width to be 73.11 nm [18]). In the first case, this distribution causes the extended exposure in the photoresist volume around the centre of the tip axis and broadens the pattern. However, since our photoresist was not very sensitive at 532 nm wavelength and because the dose radiation energy locally absorbed by the photoresist at some distance from the centre of the gaussian light profile was too small to expose those areas of photoresist, deep and narrow holes with widths of 300–340 nm and high aspect ratios of 0.22–0.26 were obtained, even with a 100 s stationary exposure time. Shorter exposure times cause the width and especially the depth of such holes to be significantly smaller [37]. A thin photoresist layer should then be used to obtain a hole pattern reaching to the substrate, so that the substrate can be etched later.

In the present studies, obtained values of aspect ratio are appreciably better compared to the values previously described in the literature [18,36,37]. Aghaei et al. [18] achieved aspect ratio of 0.08, whereas Lin et al. [37] got 0.12, which is certainly higher, but the depth of the pattern was only 3.5 nm. On the other hand, Kwon et al. [36] obtained 0.2 aspect ratio, almost equal to the one achieved in the present studies. High aspect ratio is desirable in the case of making some photonic devices, e.g., zone plates. A deep and narrow pattern allows one to obtain narrow zones in photoresist layer, which is important in the case of high resolution Fresnel zone plates, where each zone is narrower than the previous one.

### 3.3. Numerical Modeling of Focusing

In this section, modeling of laser light interaction with the structure presented in the experimental section is discussed. It is not our intention to present further experimental results of light–matter interaction using this kind of structure. We intend to show that this kind of structure is interesting from the photonics point of view for many reasons, for example, a number of adjustable parameters, like aspect ratio, length of focus, etc. The structure under consideration consists of 500 µm thick quartz substrate with an 0.7 µm thick photoresist layer with the hole pattern described in previous section. Two lines of holes are separated by 2.375 µm. The distance between holes in each line is set to 2 µm, and the diameter of each hole is 320 nm. The depth of the holes is assumed to be 77 nm. Moreover, we performed similar calculations for a structure consisting of two grooves instead of the lines of holes. The grooves have the same depth and width as the holes. Due to the periodic boundary conditions in the y direction, the length of these lines is assumed to be infinite. In the x direction, we implemented perfectly matched layers (PML) algorithm to avoid the interference with waves scattered by the patterns in adjacent unit cells.

Numerical analysis of the optical response of the structure was performed using two-dimensional (2D) multilayer RCWA [44] with implementation of the scattering matrix algorithm and the factorization rules. We employed this numerical, well convergent code previously for one-dimensional (1D) stacked metal crystals [45]. In the present case, the 2D structure approximates the 1D pattern. In the case of holes, the calculation period was taken to be 40 × 2 µm, containing one hole in y direction, thus simulating the infinitely periodic structure in the y direction. The number of diffraction orders was 225 × 13. In the case of grooves, the calculations were 1D with the calculation period in the × direction equal to 50 µm and 343 diffraction orders, which is more than sufficient to assure good convergence.

**Figure 6:**
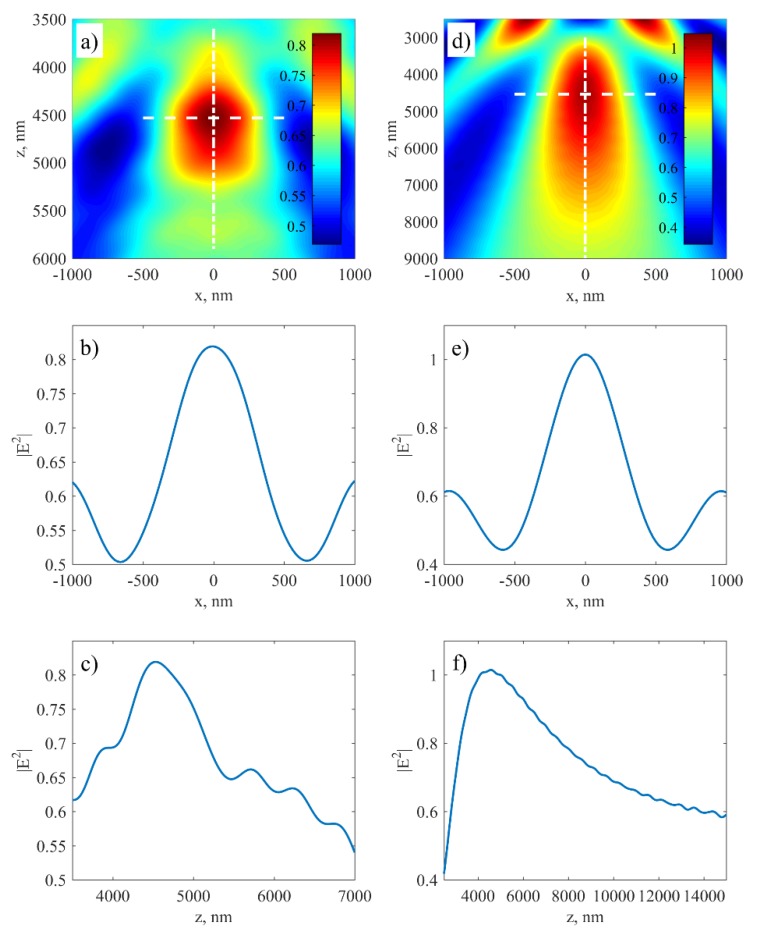
Radiation transmission through (**a**–**c**) the pattern of holes and (**d**–**f**) the pattern of grooves; (**a**,**b**) electric field intensity at y = 0; (**b**,**e**) transverse cross-sections of the focal spots along the dashed lines in (**a**,**d**); and (**c**,**f**) longitudinal cross-sections of the focal spots along the dashed-dotted lines in (**a**,**d**). The transverse cross-section of the focus in the case of hole structure is slightly larger than for grooves, but it is significantly shorter in the longitudinal direction.

Figure 6 shows the electric field intensity in the foci at y = 0 (Figure 6a,d), and their horizontal (Figure 6b,e) and longitudinal (Figure 6c,f) cross sections for hole and groove structure, respectively. The incident plane wave of 532 nm wavelength and TE (transverse-electric) polarization is impinging normally on the holes in photoresist spin-coated on quartz substrate. Refractive index of quartz is 1.4607 and of photoresist is 1.6722. It is shown that the structure can work as a zone plate and focus the plane wave into a spot. The calculated FWHM of the focus in the case of holes is around 650 nm. In the case of grooves, the FWHM is 560 nm. It is worth stressing that despite the fact that this structure consists of holes instead of straight lines and is only one-zone phase zone plate, it still can focus light on a spot comparable with a wavelength. The structure can be easily extended to contain more zones that would improve focusing properties. It is interesting that this structure shows better focusing ability than theoretically calculated Rayleigh linear resolution: res=1.22fλ/d, where *f* is focal length, *λ*—wavelength of the incident light, and *d*—diameter of the lens. Rayleigh linear resolution for a lens of the same lateral dimensions is about 2.9 µm (almost 5.5λ). Moreover, these results can be compared with a single slit-focusing experiment [46]. In the aforementioned paper, authors measured FWHM of a focal spot in case of a doubled-frequency Nd:YAG pulsed laser light impinging on a single slit in metal layer. For a slit of 1 µm width, the FWHM was equal to 420 nm. In case of wider slit (3 µm), the FWHM was 960 nm.

## 4. Conclusions

In conclusion, it was shown that SNOL has sufficient potential to be used as a method for patterning samples. The laser wavelength that lies within the less sensitive part of the spectrum allowed one to modify the experiment design. Some parameters of the structure obtained at this wavelength were better than at the maximum absorption wavelength. In addition, the use of holes instead of grooves increases the number of parameters that can be optimized and thus may improve flexibility in the design of light–matter photonic interactions. Besides, the focusing structure made of holes is characterized by producing a significantly shorter focus than that obtained in the case of grooves.

Hole array was created by using a nanoprobe of 90 nm diameter and a 532 nm laser beam over a positive photoresist. The entire experiment was carried out under standard atmospheric pressure and room temperature. However, when the sample or probe was changed, the reproducibility degraded significantly and required further optimization. Still, presented studies confirm the possibility of subwavelength patterning of the photoresist when it is not very sensitive to the laser wavelength. Optimization of width and depth of the holes was also carried out, and it was concluded that the holes with 100 s of exposure time gave satisfactory results. Further, the pattern obtained was used for its detailed characterization. It was shown that it is possible to obtain in the photoresist layer regular and uniform deep holes with high aspect ratio. Presented results were better than the data previously published in the literature [18,36,37], where the aspect ratios ranged between 0.08 [18] and 0.2 [36], while in our case they ranged from 0.22 to 0.26. In addition, the hole depths were significantly smaller than in our experiment (71–87 nm), ranging from 3.5 nm [37] to 28.3 nm [36]. However, the depth of the structure obtained still does not satisfy the conditions necessary for etching process. The experiment configuration might need to be further developed by decreasing the thickness of the photoresist and by increasing the depth of holes obtained by using higher levels of laser power.
